# Lonely Individuals Process the World in Idiosyncratic Ways

**DOI:** 10.1177/09567976221145316

**Published:** 2023-04-07

**Authors:** Elisa C. Baek, Ryan Hyon, Karina López, Meng Du, Mason A. Porter, Carolyn Parkinson

**Affiliations:** 1 Department of Psychology, University of California, Los Angeles; 2 Department of Mathematics, University of California, Los Angeles; 3 Santa Fe Institute; 4 Brain Research Institute, University of California, Los Angeles

**Keywords:** loneliness, social connection, neuroimaging, social cognition, open data, open materials

## Abstract

Loneliness is detrimental to well-being and is often accompanied by self-reported feelings of not being understood by other people. What contributes to such feelings in lonely people? We used functional MRI of 66 first-year university students to unobtrusively measure the relative alignment of people’s mental processing of naturalistic stimuli and tested whether lonely people actually process the world in idiosyncratic ways. We found evidence for such idiosyncrasy: Lonely individuals’ neural responses were dissimilar to those of their peers, particularly in regions of the default-mode network in which similar responses have been associated with shared perspectives and subjective understanding. These relationships persisted when we controlled for demographic similarities, objective social isolation, and individuals’ friendships with each other. Our findings raise the possibility that being surrounded by people who see the world differently from oneself, even if one is friends with them, may be a risk factor for loneliness.

Humans have a fundamental need to belong and connect socially (Baumeister & Leary, 1995). When this need to belong is not met, there can be devastating consequences. It is well-established that loneliness (i.e., the distressing feeling that often accompanies subjective perceptions of social disconnection) has detrimental effects on the well-being of individuals, including an increased risk of mortality that persists even after controlling for comorbidities (Holt-Lunstad et al., 2010; Shankar et al., 2011).

Feeling understood by other people is one critical factor for achieving social connection (Reis et al., 2017) and is associated with greater life satisfaction (Lun et al., 2008; Reis et al., 2017), more positive evaluations of interactions with strangers (Cross et al., 2000), and increased fulfillment in close relationships (Oishi et al., 2010). In one study, feeling understood activated brain regions that are associated with reward processing, whereas not feeling understood activated brain regions that are associated with negative affect (Morelli et al., 2013). Self-report data also suggest that there is an association between loneliness and not feeling understood by other individuals (Routasalo et al., 2006). Such findings suggest that not feeling understood by other people may be a risk factor for loneliness. However, it is unknown whether lonely people actually see the world in ways that are dissimilar to others in their community (rather than, e.g., exaggerating how dissimilar others’ views are to their own), which may contribute to feelings of disconnection due to a lack of shared understanding.

We used neuroimaging to test the hypothesis that lonely^1^ people have neural responses to naturalistic stimuli (specifically, videos) that are idiosyncratic in comparison to those of their peers (including other lonely people), perhaps contributing to the lack of feeling understood that often accompanies loneliness. Measuring brain responses during a naturalistic paradigm in which people view audiovisual stimuli that unfold over time provides a window into individuals’ unconstrained thought processes as they develop and evolve (Sonkusare et al., 2019). Additionally, examining the similarity of neural responses can simultaneously capture various types of processing similarity, including similarities in high-level interpretations and understanding (Nguyen et al., 2019; Yeshurun et al., 2017, 2021), affective processing (Nummenmaa et al., 2012), and patterns of attention allocation (e.g., mind wandering vs. paying attention; Song et al., 2021; the aspects of stimuli to which people attend; Lahnakoski et al., 2014). Accordingly, the extent to which an individual exhibits similar neural responses to their peers can provide insights into the extent to which they process the world in a way that is similar to their peers.

Much work that uses intersubject correlations (ISCs) of neural responses has looked at whether and where similarities in behavior, interpretive frames and expectations, or traits are associated with similarities in neural responding (Nguyen et al., 2019; Yeshurun et al., 2017). Other approaches have calculated ISCs to examine how people’s overall levels of particular traits or symptoms relate to their level of attunement with others (Bolis et al., 2017). Accordingly, we tested the following hypothesis: Nonlonely people are all alike, but every lonely individual processes the world in their own idiosyncratic way. In other words, we tested whether the associations between loneliness and neural responses to naturalistic stimuli follow an “Anna Karenina principle.” This principle is inspired by the opening line from the novel *Anna Karenina*: “Happy families are all alike; every unhappy family is unhappy in its own way” (Tolstoy, 1997). It proposes that successful endeavors are marked by similar characteristics but that unsuccessful endeavors are each different in their own idiosyncratic way (Diamond, 1997). Studying various phenomena in light of this principle has yielded a variety of meaningful insights (Diamond, 1997; Finn et al., 2018). In the present study, we tested whether individuals who are not lonely are exceptionally similar to each other in how they process the world, whereas lonely individuals each process the world in their own distinct way.

We focused on subjective social isolation (i.e., individuals’ perceptions or feelings of isolation), but objective social isolation has also been linked to myriad negative health outcomes (Shankar et al., 2011). Objective social isolation is often measured by obtaining information about an individual’s self-reported social-network size (Moieni & Eisenberger, 2020). Although objective and subjective social isolation are associated with each other, they are distinct constructs (Moieni & Eisenberger, 2020; Routasalo et al., 2006), and some evidence suggests that subjective feelings of social isolation are linked more strongly than objective social isolation to negative health outcomes (Holwerda et al., 2014). To disentangle people’s perceptions of their objective levels of social connection/disconnection from their subjective feelings of social connection/disconnection, we conducted additional analyses that account for objective social isolation by controlling for the number of friends (i.e., out-degree centrality) that individuals reported having in their communities. This approach allowed us to test whether individuals who experience high levels of loneliness have neural responses that are dissimilar to those of their peers and to each other, even after controlling for their objective numbers of social ties. In other words, we tested whether lonely individuals process the world idiosyncratically, even if they have many friends.

**Statement of Relevance**

Loneliness (i.e., the distressing feeling that often accompanies subjective perceptions of social disconnection), which is pervasive and consequential, can have devastating consequences on both mental and physical well-being. Prior work suggests that lonely individuals self-report not feeling understood by other people. Given the importance of feeling understood in achieving social connection, the feeling of not being understood by other individuals may be one feature that characterizes loneliness. What contributes to such feelings in lonely individuals? In this study, we used neuroimaging to show that lonely individuals process the world in idiosyncratic ways that are exceptionally dissimilar to their peers. Such idiosyncratic neural processing may contribute to feelings of disconnection from a lack of shared understanding. Our findings elucidate the underlying processes that characterize loneliness.

## Method

### Functional MRI study participants

**Fig. 1.**
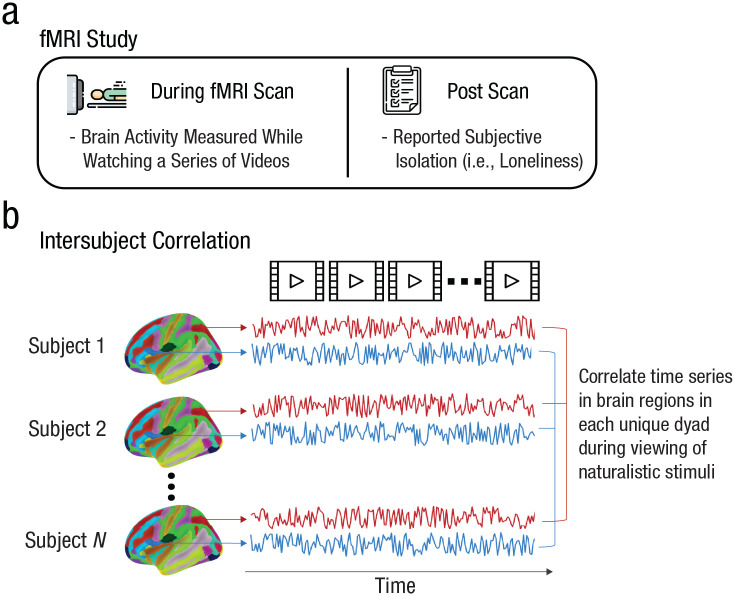
Overview of the study paradigm and analysis. (a) Schematic of the study paradigm. Participants attended an in-lab session in which their brain activity was measured using functional MRI (fMRI) while they watched a series of naturalistic stimuli (specifically, videos). After the scan, participants completed the UCLA Loneliness Scale (ULS-8; Hays & Dimatteo, 1987). (b) Schematic of the analysis. We extracted time series of neural responses to the stimuli in each of 214 brain regions, and we then correlated these time series across participants to calculate intersubject correlations for each dyad in each brain region.

A total of 70 individuals from two residential communities participated in our neuroimaging study, which consisted of a functional MRI session and self-report questionnaires (see Fig. 1). We selected two residential communities to obtain a sample size in line with those in other neuroimaging studies with similar paradigms that relate neural similarity to behavioral traits (Finn et al., 2018; Nguyen et al., 2019; Parkinson et al., 2018). We finished collecting data after scanning all interested members of these communities. The participants in our study consisted of first-year students at a large public university (University of California, Los Angeles) in the United States. We excluded four participants from the functional MRI data because two of them had excessive movement in more than half of the scan, one participant fell asleep during half of the scan, and one participant did not complete the scan. This resulted in a total of 66 participants (41 female) between the ages of 18 and 21 years (mean = 18.23 years, *SD* = 0.63) that we included in our primary analyses of the relationship between ISCs and loneliness. Of these subjects, one participant had excessive head movement in one of the four runs and one participant reported falling asleep in one of the four runs. We excluded the respective runs for these participants and included only the remaining three runs for these participants in analyses that involved brain data. All participants provided informed consent in accordance with the procedures of the Institutional Review Board of the University of California, Los Angeles. We reported on separate analyses of the same data set in other articles to investigate associations between neural similarity and (a) aspects of individuals’ positions in their social networks (Baek et al., 2022) and (b) personality similarity (Matz et al., 2022). In the present study, we investigated associations between neural similarity and individuals’ subjective feelings of social disconnection.

### Functional MRI procedure

Participants attended an in-person study session that included self-report surveys and a 90-min neuroimaging session in which their brain activity was measured using blood-oxygen-level-dependent (BOLD) functional MRI. The functional MRI data collection occurred between September 2019 and early November 2019 during the participants’ first year at the university and prior to the social-network survey. (See the Characterizing Subjective and Objective Social Disconnection section.) Prior to entering the scanner, participants completed self-report surveys in which they provided demographic information such as their age, gender, and race/ethnicity. During the functional MRI portion of the study, the participants watched 14 different video clips with sound. These 14 videos ranged in duration (91–734 s) and content. (For descriptions of the content, see Table S1 in the online Supplemental Material.) Prior to the scanning session, participants were informed that they would be watching video clips of heterogeneous content and that their experience would be akin to watching television while someone else “channel surfed.”^2^ We selected a subset of the video clips from ones that had been used previously, and we used similar criteria to those in prior works to select new stimuli (Hyon et al., 2020; Parkinson et al., 2018). First, to avoid inducing intersubject differences from heterogeneous familiarities with content, we selected stimuli that were unlikely to have been seen before by our participants. Second, to minimize the likelihood that participants would engage in mind wandering during viewing (because that could introduce undesirable noise into our data), we selected stimuli that were likely to be engaging. Third, we selected stimuli that were likely to elicit meaningful variability in interpretations and meaning that different individuals would draw from the content. Participants were asked to watch the videos naturally (i.e., as they would watch them in a normal situation in life). All participants saw the videos in the same order to avoid any potential variability in neural responses from differences in the way that the stimuli were presented (rather than from endogenous participant-level differences). The video “task” was divided into four runs, and each run consisted of a continuous stream of content. In each run, the video clips were presented immediately after one another, with no gap between the clips. The task lasted approximately 60 min in total. Structural images of the brain were also collected. For more details, see the Functional MRI Data Acquisition section.

### Characterizing subjective and objective social disconnection

To characterize the participants’ subjective feelings of social disconnection, we administered the UCLA Loneliness Scale (ULS-8; Hays & Dimatteo, 1987) on the day of each participant’s functional MRI scan. We based our characterizations of participants’ objective levels of social disconnection on their responses to a separate social-network survey that was administered during December and January of the students’ first year at the university. (The academic year began at the end of September.) In the survey, participants were asked to type the names of other residents in their residential community with whom they interacted regularly. They were prompted with the following question: “Consider the people you like to spend your free time with. Since you arrived at [institution name], who are the people you’ve socialized with most often? (Examples: eat meals with, hang out with, study with, spend time with).” The participants were free to name as many people as they desired without any restrictions, and no time limit was imposed. This question was adapted from prior research that investigated the social networks of university students (Burt, 2004; Parkinson et al., 2018). Using the participants’ answers to this question, we calculated out-degree centrality, which is equal to the number of the participants’ community members with whom they reported socializing regularly. This allowed us to obtain an inverse measure of participants’ objective levels of social disconnection (i.e., high out-degree centrality values reflect low levels of social disconnection and low out-degree centrality values reflect high levels of social disconnection), which we then used as a control variable.

A total of 119 subjects completed the social-network survey; 66 of these participants also participated in the functional MRI session. Of these 66 participants, two participants were excluded because of excessive head movement and one participant was excluded because they fell asleep. This resulted in a total of 63 participants (40 female) that we included for all analyses that incorporated out-degree centrality as a control variable.

### Functional MRI data acquisition

Participants were scanned using a 3T Siemens Prisma scanner with a 32-channel head coil (Siemens Medical Solutions, Malvern, PA). Functional images were recorded using an echo-planar sequence (echo time = 37 ms, repetition time = 800 ms, voxel size = 2.0 mm × 2.0 mm × 2.0 mm, matrix size = 104 mm × 104 mm, field of view = 208 mm, slice thickness = 2.0 mm, multiband acceleration factor = 8, and 72 interleaved slices with no gap). A black screen was included at the beginning (duration = 8 s) and the end (duration = 20 s) of each run to allow the BOLD signal to stabilize. We also acquired high-resolution T1-weighted images (echo time = 2.48 ms, repetition time = 1,900 ms, voxel size = 1.0 mm × 1.0 mm × 1.00 mm, matrix size = 256 mm × 256 mm, field of view = 256 mm, slice thickness = 1.0 mm, and 208 interleaved slices with a 0.5-mm gap) for coregistration and normalization. We attached adhesive tape to the head coil in the MRI scanner and applied it across participants’ foreheads; this method significantly reduces head motion (Krause et al., 2019). We used fMRIPrep (Version 1.4.0) for the data processing of our functional MRI data (Esteban et al., 2019). See the Supplemental Material for technical details about data preprocessing.

### Cortical parcellation

We extracted neural responses across the whole brain using the 200-parcel cortical parcellation scheme of Schaefer et al. (2018) along with 14 subcortical parcels using the Harvard–Oxford subcortical atlas (Desikan et al., 2006). This gave a total of 214 parcels.

### Intersubject correlations

We extracted preprocessed time-series data and concatenated all four runs into a single time series for each participant, except for the two participants for whom we used only partial data. For these two participants, we concatenated their three usable runs into a single time series and calculated ISCs for these participants by comparing their data with the corresponding three runs in the other participants. We averaged the response time series across the voxels within each of the 214 brain regions for each participant at each repetition time. Our main analyses included 66 participants, so there were 2,144 unique dyads in these analyses. Our analyses that included out-degree centrality as a control variable had 63 participants, so there were 1,952 unique dyads for these analyses. For each unique dyad, we calculated the Pearson correlation between the mean time series of the neural response in each of the 214 brain regions. We then took Fisher *z* transforms of the Pearson correlations and normalized the subsequent values (i.e., we “*z*-scored” them) in each brain region prior to our analyses.

### Relating ISC with loneliness

We took the following steps to test for associations between loneliness and neural similarity in each of the 214 brain regions. First, we used a median split to stratify our sample into lonely and nonlonely groups. For conciseness, we refer to people with a ULS-8 score above the median as “lonely” and to people with a ULS-8 score at or below the median as “nonlonely.” Our choice to use a median split follows the example of other recent studies that related neural similarity with behavioral measures (Finn et al., 2018; Leong et al., 2020). Whenever possible, we also conducted exploratory tests to investigate relationships between ISCs and the nonbinarized loneliness variable. We describe this in more detail below.

To relate the dyad-level neural similarity measure with loneliness, we transformed the individual-level binarized loneliness measure into a dyad-level variable. We labeled dyads as (1) {lonely, lonely} if both individuals in a dyad were lonely, (2) {nonlonely, nonlonely} if both individuals in a dyad were nonlonely, and (3) {nonlonely, lonely} if one individual in a dyad was nonlonely and the other individual was lonely. To relate this dyad-level loneliness measure to neural similarity, we used the method of Chen et al. (2017) and fit linear mixed-effects models with crossed random effects using lme4 and lmerTest in the R programming environment (Kuznetsova et al., 2017; R Core Team, Version 3.6.1). This approach allowed us to account for nonindependence in the data from repeated observations for each participant (i.e., because each participant is part of multiple dyads). Following the method that was suggested by Chen et al. (2017), we “doubled” the data (with redundancy) to allow fully crossed random effects. In other words, we accounted for the symmetric nature of the ISC matrix and the fact that each participant contributes twice in a dyad (because (*i*, *j*) = ( *j*, *i*) for participants *i* and *j*). For more details, including information about the approach and terminology, see Chen et al. (2017). Before performing statistical inference, we manually corrected the degrees of freedom to *N - k*, where *N* is the number of unique observations (in our case, *N* = 2,144) and *k* is the number of fixed effects in the model. All findings that we report in the present article use the corrected number of degrees of freedom. For each of our 214 brain regions, we first fit a mixed-effects model, with ISCs in the corresponding brain region as the dependent variable, the dyad-level binarized loneliness variable as the independent variable, and random intercepts for each individual (i.e., “Participant 1” and “Participant 2”) in a dyad. We then performed planned contrasts using emmeans in the R programming environment (Russell et al., 2021) to identify the brain regions where the inclusion of even one lonely individual is associated with smaller ISCs: ISC_{lonely, lonely}_ > ISC_{nonlonely, nonlonely}_, ISC_{lonely, lonely}_ > ISC_{nonlonely, lonely}_, and ISC_{nonlonely, lonely}_ > ISC_{nonlonely, nonlonely}_. We *z*-scored all variables to yield standardized coefficients (β) as outputs. We false-discovery-rate corrected (FDR-corrected) *p* values for multiple comparisons at *p* < .05.

For our exploratory analysis in which we related a nonbinarized version of the loneliness variable to ISCs, we used the maximum loneliness value of each dyad. For instance, if Participant 1 in a dyad had a loneliness value of 4 and Participant 2 in that dyad had a loneliness value of 6, then we assigned a loneliness value of 6 to the dyad. The choice of taking the maximum loneliness value of each dyad allowed us to test the hypothesis that only dyads with two nonlonely individuals have very similar neural responses to each other. If loneliness is associated with idiosyncratic neural responses, then the inclusion of even just one lonely individual in a dyad should be associated with low ISCs. We repeated the procedure that we described above to fit mixed-effects models with fully crossed random effects to infer the ISCs in each brain region from the dyadic maximum loneliness. In other words, for each of the 214 brain regions, we fit a mixed-effects model, with ISCs in the corresponding brain region as the dependent variable, the maximum loneliness value of the dyad as the independent variable, and random intercepts for each individual (i.e., “Participant 1” and “Participant 2”) in a dyad. We *z*-scored all variables, and we FDR-corrected all *p* values for multiple comparisons at *p* < .05.

### Relating ISC with loneliness while controlling for overall levels of objective social disconnection, friendships between participants, and demographic similarities

We fit additional models to test whether any observed associations between loneliness and ISCs remain significant even after controlling for participants’ self-reported levels of objective social disconnection, whether the individuals in a dyad were friends (because prior research suggests that friends have larger ISCs than nonfriends; Hyon et al., 2020; Parkinson et al., 2018), and dyadic similarities in all available demographic variables. (For details about how we calculated these variables, see the Supplemental Material.) To do this, we fit linear mixed-effects models with fully crossed random effects using the method of Chen et al. (2017), as described in the Relating ISC With Loneliness section in the Method section, with ISC in the corresponding brain region as the dependent variable and the dyad-level loneliness variable as the independent variable of interest, while controlling for out-degree centrality; friendships between individuals in a dyad; and dyadic similarities in age, gender, race/ethnicity, and home country by including them as covariates of no interest. We then performed planned contrasts (i.e., ISC_{lonely, lonely}_ > ISC_{nonlonely, nonlonely}_, ISC_{lonely, lonely}_ > ISC_{nonlonely, lonely}_, and ISC_{nonlonely, lonely}_ > ISC_{nonlonely, nonlonely}_) to test whether the inclusion of one or more highly lonely individuals in a dyad is associated with smaller ISCs while controlling for the aforementioned variables.

## Results

### Associations between binarized loneliness and neural similarity

We first tested whether loneliness is associated with more idiosyncratic brain activity than one’s peers. The loneliness scores in our data ranged from a minimum of 8 to a maximum of 27 (with a possible minimum of 8 and a possible maximum of 32), with a mean of 15.91, a median of 16, a mode of 16, and a standard deviation of 4.879. Our median-split approach identified 35 individuals who had a loneliness score above the median and 31 individuals who had a loneliness score at or below the median. For a plot of the distribution of the loneliness scores, see Figure S1 in the Supplemental Material. To relate the dyad-level neural similarity measure with loneliness, we then transformed the individual-level binarized loneliness measure into a dyad-level variable. (For more details, see the Relating ISC With Loneliness section in the Method section.) Of the 2,144 unique dyads, 595 were {lonely, lonely}, 465 were {nonlonely, nonlonely}, and 1,084 were {nonlonely, lonely}.

**Fig. 2.**
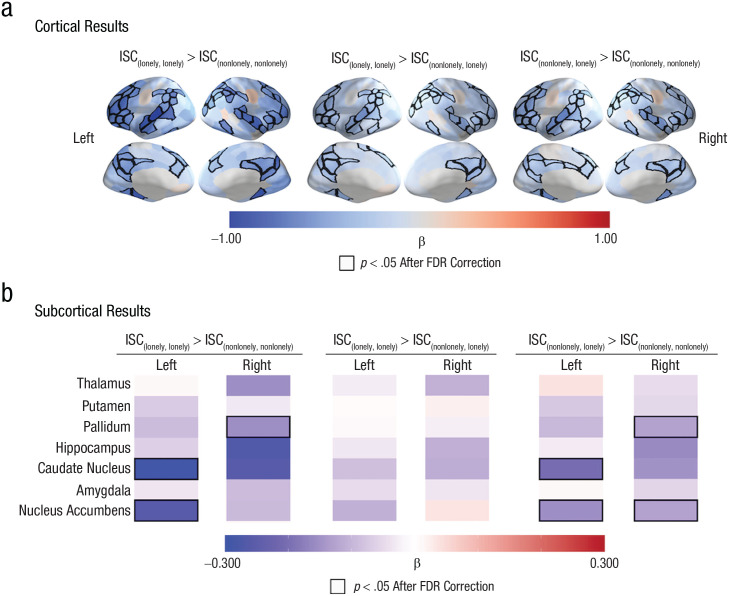
Linking greater levels of loneliness to more idiosyncratic neural responses. (a) The intersubject correlations (ISCs) were smaller in brain regions such as the dorsal medial prefrontal cortex, precuneus, ventrolateral prefrontal cortex, dorsal lateral prefrontal cortex, superior temporal sulcus, inferior parietal lobule, and superior parietal lobule in dyads in which both individuals were lonely (i.e., {lonely, lonely}) than in dyads in which neither individual was lonely (i.e., {nonlonely, nonlonely}). We observed similar patterns when we compared dyads in which both individuals were lonely (i.e., {lonely, lonely}) with dyads with one nonlonely individual and one lonely individual (i.e., {nonlonely, lonely}) and when we compared dyads with one nonlonely individual and one lonely individual (i.e., {nonlonely, lonely}) with dyads with two nonlonely individuals (i.e., {lonely, lonely}). (b) The ISCs were smaller in the right pallidum, left caudate nucleus, and left nucleus accumbens in dyads with two lonely individuals than in dyads with two nonlonely individuals. The labels “Left” and “Right” refer to the hemispheres of the brain regions that are listed in the left panel. The quantity β is the standardized regression coefficient. Regions with significant associations between loneliness and ISC are outlined in black; a false-discovery-rate corrected (FDR-corrected) significance threshold of *p* < .05 was used.

We used planned contrasts to compare dyadic neural similarities across the different loneliness groups and to identify brain regions where the inclusion of one or more lonely individuals in a dyad is associated with less-coordinated neural responses (i.e., ISC_{lonely, lonely}_ > ISC_{nonlonely, nonlonely}_, ISC_{lonely, lonely}_ > ISC_{nonlonely, lonely}_, and ISC_{nonlonely, lonely}_ > ISC_{nonlonely, nonlonely}_). We found that dyads in which both individuals were lonely had smaller ISCs than dyads in which both members were nonlonely (see Fig. 2 and Tables S2–S4 in the Supplemental Material) in the dorsal medial prefrontal cortex, ventrolateral prefrontal cortex, dorsal lateral prefrontal cortex, precuneus, posterior cingulate cortex, superior temporal cortex (including the superior temporal sulcus), superior parietal lobule, and inferior parietal lobule. The ISCs in several subcortical regions—specifically, the left nucleus accumbens, left caudate nucleus, and right pallidum—were also smaller in {lonely, lonely} dyads than in {nonlonely, nonlonely} dyads. See Figure 2 and Tables S5 and S6 in the Supplemental Material.

### Associations between nonbinarized loneliness and neural similarity

We also related mean ISCs to a nonbinarized loneliness value (specifically, the maximum loneliness value in a dyad, as we discussed in the Relating ISC With Loneliness section in the Method section). As with our results with the binarized loneliness variable, we found a negative association between maximum loneliness and neural similarity in the ventrolateral prefrontal cortex, dorsal lateral prefrontal cortex, precuneus, posterior cingulate cortex, superior temporal cortex, inferior parietal lobule, and superior parietal lobule. In other words, mirroring our results for the binarized loneliness measure, dyads in which one or both individuals were lonely (as indicated by a higher maximum loneliness value) were characterized by less neural similarity than dyads in which both individuals were nonlonely (as indicated by a lower maximum loneliness value). See Figure S2 in the Supplemental Material.

**Fig. 3.**
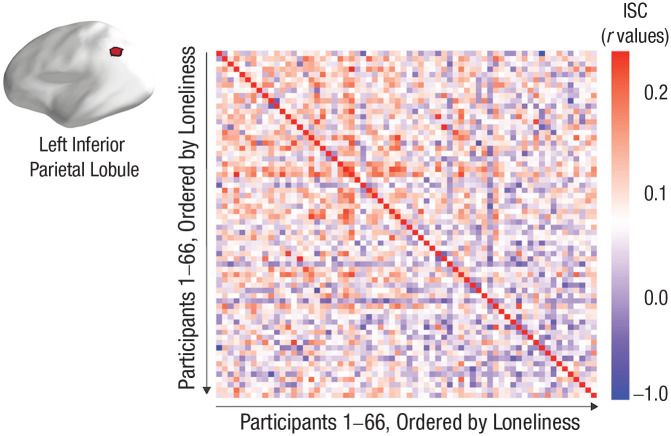
Associations between intersubject correlations (ISCs) and loneliness in the left inferior parietal lobule. The ISC matrix is for a parcel in the left inferior parietal lobule; the rows and columns of the matrix are ordered by increasing loneliness. The largest ISC values tend to occur in the top-left corner of the matrix (as indicated by warm colors). This corner has the dyads with the lowest loneliness scores. The bottom-right portions of the matrix tend to have the smallest ISC values (as indicated by cool colors). The pattern of results in this matrix supports the hypothesis that having at least one lonely person in a dyad is associated with smaller ISCs.

In Figure 3, we show the relationship between loneliness and ISC in a parcel in the left inferior parietal lobule, in which neural similarity has been associated previously both with shared understanding of events (Nguyen et al., 2019; Yeshurun et al., 2017) and with similarities in perspectives (Lahnakoski et al., 2014). This figure shows the participant-by-participant ISC matrix for this parcel, with rows and columns ordered by participants’ loneliness scores. The ISCs tend to become smaller as one moves to the right and down, indicating that the smallest ISCs tend to occur in increasingly lonely dyads.

### Associations between loneliness and neural similarity when controlling for objective social disconnection, friendships between participants, and demographic similarities

**Fig. 4.**
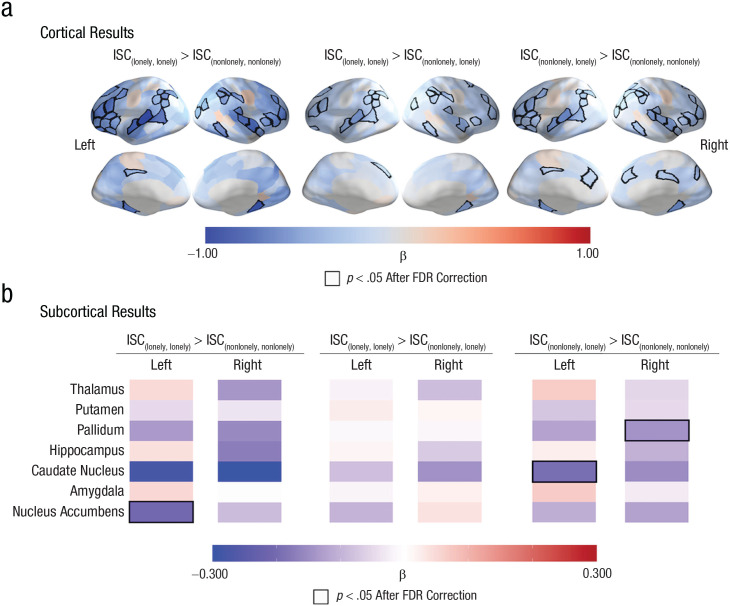
Linking loneliness to idiosyncratic neural responses while controlling for objective social disconnection, demographic similarities, and friendships between participants. (a) As in our main results, intersubject correlations (ISCs) were smaller in brain regions (including the ventrolateral prefrontal cortex, dorsal lateral prefrontal cortex, superior temporal sulcus, inferior parietal lobule, and superior parietal lobule) that are associated with social cognition, shared understanding of events, and friendship in dyads with individuals who were both lonely (i.e., {lonely, lonely}) than in dyads in which both individuals were nonlonely (i.e., {nonlonely, nonlonely}). We observed similar patterns when we compared dyads with individuals who were both lonely (i.e., {lonely, lonely}) with dyads with one nonlonely individual and one lonely individual (i.e., {nonlonely, lonely}) and when we compared dyads with one nonlonely individual and one lonely individual (i.e., {nonlonely, lonely}) with dyads with two nonlonely individuals (i.e., {nonlonely, nonlonely}). (b) The ISCs were smaller in the left nucleus accumbens in dyads with two lonely individuals than in dyads with two nonlonely individuals. The labels “Left” and “Right” refer to the hemispheres of the brain regions that are listed in the left panel. The quantity β is the standardized regression coefficient. Regions with significant associations between loneliness and ISC are outlined in black; a false-discovery-rate corrected (FDR-corrected) significance threshold of *p* < .05 was used.

We also tested whether the associations between loneliness and ISCs remain significant even after controlling for participants’ self-reported levels of objective social disconnection, whether the individuals in a dyad were friends, and dyadic similarities in all available demographic variables. As in our main results, we found that ISCs in the ventrolateral prefrontal cortex, dorsal lateral prefrontal cortex, superior temporal cortex, superior parietal lobule, and inferior parietal lobule were smaller in dyads in which both individuals were lonely ({lonely, lonely}) than in dyads in which both individuals were not lonely ({nonlonely, nonlonely}). See Figure 4a and Table S7 in the Supplemental Material. Additionally, ISCs in the left nucleus accumbens were smaller in {lonely, lonely} dyads than in {nonlonely, nonlonely} dyads. See Figure 4b and Table S8 in the Supplemental Material. We observed similar patterns when we compared {lonely, lonely} dyads with {nonlonely, lonely} dyads and {nonlonely, lonely} dyads with {nonlonely, nonlonely} dyads. See Figure 4b and Tables S9–S11 in the Supplemental Material.

Given recent findings that link neural similarity with popularity (Baek et al., 2022), we also fit models that control for participants’ in-degree centrality (i.e., the number of times that participants were nominated as a friend by others in their social network). The results of these analyses (see Fig. S3 in the Supplemental Material) are similar to those of our main analyses.

## Discussion

Our results suggest that lonely people process the world idiosyncratically, which may contribute to the reduced sense of being understood that often accompanies loneliness. In other words, we found that nonlonely individuals were very similar to each other in their neural responses, whereas lonely individuals were remarkably dissimilar to each other and to their nonlonely peers. Our results remained significant even after controlling for individuals’ objective levels of social connection/disconnection (specifically, their numbers of friends), demographic variables, and friendships between participants. Therefore, we conclude that lonely people may view the world in a way that is different from their peers. These findings raise the possibility that being surrounded predominantly by people who view the world differently from oneself may be a risk factor for loneliness (even if one socializes regularly with them).

Brain areas in which responses were more similar include the dorsal medial prefrontal cortex, precuneus, ventrolateral prefrontal cortex, dorsal lateral prefrontal cortex, superior temporal sulcus, superior parietal lobule, inferior parietal lobule, and nucleus accumbens. Notably, in many of these regions—in particular, those that belong to the default-mode network—similar neural responding when watching videos has been associated with similarities in understanding and interpretation of narratives (Finn et al., 2018; Nguyen et al., 2019; Yeshurun et al., 2017) and friendship (Parkinson et al., 2018). We found that this pattern remained similar even after controlling for objective social connection/disconnection, demographic similarities, and friendships between participants. Consequently, our results suggest that lonely individuals process the world in a way that is dissimilar to their peers and to each other. Future work can further test this possibility by using behavioral experimentation and semantic analyses to examine what aspects of lonely individuals’ interpretations are particularly idiosyncratic.

Although it is unclear whether the observed idiosyncratic processing in lonely individuals is a cause or a result of loneliness, the associated lack of shared understanding may lead to challenges in achieving social connections. These effects hold even after controlling for the number of friends of individuals and whether two individuals in a dyad are friends with each other, suggesting that our findings are not merely a consequence of lonely people being less likely to have friends or nonlonely individuals being more likely to be friends with each other. Instead, we observed that individuals with high levels of loneliness—regardless of the number of their objective social connections—were more likely to have idiosyncratic neural responses. It is also likely that the extent of objective and subjective social connection/disconnection fluctuates with time, which may in turn influence or be influenced by the extent to which an individual processes the world idiosyncratically. Future work that implements a longitudinal design may further elucidate the causal directions of these relationships. Furthermore, in our study, we focused on subjective feelings of social disconnection and thus on individuals’ self-reported perceptions of social disconnection. Future work that complements self-reported measures of social disconnection with measures of relevant affective and behavioral phenomena (e.g., based on structured interviews or on the reports of peer or family members) may further enrich our understanding of these phenomena.

In our study, we collected the data for characterizing subjective and objective social connection/disconnection during two different time periods. The gap occurred during a particularly important transitional period in individuals’ lives (specifically, participants’ first few months at a university). We obtained the functional MRI data and the loneliness measures between September and November, whereas we collected the social-network survey data in December and January. Although the time gap between the two data collections was small, future work that tests the relationships between neural similarity, subjective social connection/disconnection, and objective social connection/disconnection measures that are obtained simultaneously and/or in different social contexts can enrich our understanding of how these variables interact and change with time. Additionally, our study sample consisted of university students who were young adults (specifically, between ages 18 and 21 years), and roughly two thirds of our participants were female. Future work on the relationships between loneliness and neural similarity in nonuniversity communities can help elucidate how much the relationships that we observed in the present study generalize to other populations and social contexts.

Our findings dovetail with recent suggestions that the default-mode network is a “sense-making” network that combines external information about an individual’s environment with existing internal information of their past memories and knowledge (Yeshurun et al., 2021) and findings that suggest that loneliness is associated with structural and functional differences in the default-mode network (Spreng et al., 2020). For instance, loneliness has been associated with greater variation in gray-matter volume in the default-mode network, suggesting that there is greater idiosyncrasy in the structure of the default-mode network in lonely individuals than in nonlonely individuals. We found that lonely individuals have idiosyncratic functional brain responses to audiovisual stimuli in regions of the default-mode network, whereas nonlonely individuals were exceptionally similar to each other. Our findings thereby add further insight into the idiosyncrasies of the default-mode network that may characterize lonely individuals.

What types of idiosyncratic thought processes characterize lonely individuals? Prior research offers some clues about what thought processes may potentially lead to idiosyncrasies in neural responses in lonely individuals. In our study, lonely individuals had smaller ISCs in subcortical regions (such as the left nucleus accumbens) that constitute part of the brain’s reward system (Knutson et al., 2001) and in regions of the lateral posterior parietal cortex that are associated with bottom-up and top-down orienting of attention (Corbetta & Shulman, 2002). Therefore, one possibility is that lonely individuals do not find value in the same aspects of situations or scenes as their peers (and instead focus on other aspects of situations in an idiosyncratic fashion), perhaps because of differences in their preferences, expectations, and/or memories that can in turn shape how they attend to and interpret stimuli. This may result in a reinforcing feedback loop in which lonely individuals perceive themselves to be different from their peers, which may in turn lead to further challenges in achieving social connection. Indeed, in one recent study (Courtney & Meyer, 2020), greater loneliness was associated with reduced neural representational similarity between oneself and other people in the medial prefrontal cortex, suggesting that lonely individuals think of themselves in a way that is more dissimilar to others than is the case for nonlonely individuals. Exceptional dissimilarities between lonely individuals and their peers in how they process the world may contribute to an overall sense of lacking shared understanding that often accompanies loneliness.

In summary, our findings suggest that processing the world differently from people around oneself is linked to loneliness. These findings were reflected in lonely individuals’ idiosyncratic neural responses in brain regions that have been associated with shared interpretations of events, attentional orienting, and reward processing. Moreover, these effects remained significant even after controlling for objective social disconnection and friendships between individuals. Therefore, being surrounded by people who view the world differently from oneself may be a risk factor for loneliness, even if one socializes regularly with them.

## Supplemental Material

sj-docx-1-pss-10.1177_09567976221145316 – Supplemental material for Lonely Individuals Process the World in Idiosyncratic Ways Click here for additional data file. Supplemental material, sj-docx-1-pss-10.1177_09567976221145316 for Lonely Individuals Process the World in Idiosyncratic Ways by Elisa C. Baek, Ryan Hyon, Karina López, Meng Du, Mason A. Porter and Carolyn Parkinson in Psychological Science

## Transparency

*Action Editor:* Daniela Schiller

*Editor:* Patricia J. Bauer

*Author Contribution(s)*

**Elisa C. Baek:** Conceptualization; Data curation; Formal analysis; Investigation; Methodology; Visualization; Writing – original draft; Writing – review & editing.

**Ryan Hyon:** Conceptualization; Investigation; Methodology; Writing – review & editing.

**Karina López:** Investigation; Project administration; Writing – review & editing.

**Meng Du:** Conceptualization; Data curation; Methodology; Writing – review & editing.

**Mason A. Porter:** Conceptualization; Funding acquisition; Supervision; Writing – review & editing.

**Carolyn Parkinson:** Conceptualization; Funding acquisition; Methodology; Supervision; Writing – review & editing.

**Open Practices:** The custom code that we used for the study is available at https://zenodo.org/record/7105933. The data are available at https://zenodo.org/record/7186901. The stimuli that we used in the study are available at https://gitlab.com/anon_authors/dorm_study_stimuli. The study was not preregistered.

## Notes

1. For more details about how we classified participants as lonely, see the Relating ISC With Loneliness section in the Method section.

2. The term “channel surfing” is an idiom that refers to scanning through different television channels.
