# Supplementary material for: Lonely Individuals Process the World in Idiosyncratic Ways
Source: Psychol Sci. 2023 Apr 7;34(6):683–95. doi: 10.1177/09567976221145316 (PMC10404901; doi:10.1177/09567976221145316)
Supplement: sj-docx-1-pss-10.1177_09567976221145316 – Supplemental material for Lonely Individuals Process the World in Idiosyncratic Ways [file sj-docx-1-pss-10.1177_09567976221145316.docx]

Supplemental Materials for “**Lonely individuals process the world in idiosyncratic ways”**

**Supplementary method**

**fMRI data analysis.** We used fMRIPrep version 1.4.0 to process our fMRI data (Esteban et al., 2019). We have taken the descriptions of anatomical and functional data preprocessing in this section from the recommended boilerplate text that is generated by fMRIPrep and released under a CC0 license, with the intention that researchers reuse the text to facilitate clear and consistent descriptions of preprocessing steps (and thereby enhance the reproducibility of studies).

For each participant, the T1-weighted (T1w) image was corrected for intensity non-uniformity (INU) with N4BiasFieldCorrection, distributed with ANTs 2.1.0 (Avants et al., 2011), and used as T1w-reference throughout the workflow. Brain tissue segmentation of cerebrospinal fluid (CSF), white matter (WM), and gray matter (GM) was performed on the brain-extracted T1w using FSL fast (Smith et al., 2004). Volume-based spatial normalization to the ICBM 152 Nonlinear Asymmetrical template version 2009c (MNI152NLin2009cAsym) was performed through nonlinear registration with antsRegistration (ANTs 2.1.0; Avants et al., 2011).

For each of the BOLD runs of each participant, the following preprocessing was performed. First, a reference volume and its skull-stripped version were generated using a custom methodology of fMRIPrep. The BOLD reference was then coregistered to the T1w reference using FSL flirt (Smith et al., 2004) with the boundary-based registration cost function. Coregistration was configured with nine degrees of freedom to account for distortions remaining in the BOLD reference. Head-motion parameters with respect to the BOLD reference (transformation matrices, and six corresponding rotation and translation parameters) were estimated before any spatiotemporal filtering using FSL mcflirt (Smith et al., 2004). Automatic removal of motion artifacts using independent component analysis (ICA-AROMA) was performed on the preprocessed BOLD on MNI-space time series after removal of non-steady-state volumes and spatial smoothing with an isotropic, Gaussian kernel of 6mm FWHM (full-width half-maximum). The BOLD time series were then resampled to the MNI152Nlin2009cAsym standard space.

The following 10 confounding variables generated by fMRIPrep were included as nuisance regressors: global signals extracted from within the cerebrospinal fluid, white matter, and whole-brain masks; framewise displacement; three translational motion parameters; and three rotational motion parameters.

**Calculation of control variables.** We took an analogous approach to the one that we described in “Relating ISC with loneliness” section in the Method section of the main manuscript to transform the individual-level out-degree centrality variable into a dyad-level variable to control for objective social disconnection. First, using each participant’s response to the social-network survey, we calculated their out-degree centrality (i.e., the number of people in their residential community that the participant nominated as a friend). As in our approach in “Relating ISC with loneliness” section in the Method section of the main manuscript, we used a median split to label each individual as having a “high” or “low” level of objective social disconnection. We categorized participants into the low objective social-disconnection group if they had an out-degree that was larger than the median and into the high objective social-disconnection group if they had an out-degree that was less than or equal to the median. We then transformed the individual-level out-degree centrality variable into a dyad-level variable using the method that we described in “Relating ISC with loneliness” section in the Method section of the main manuscript to transform the individual-level out-degree variable into a dyad-level variable.

To control for similarities in age, for each dyad, we computed the absolute value of the difference between the ages of the two individuals in the dyad (i.e., age_difference = |age_participant_1_  – age_participant_2_|). We then transformed this difference into a similarity measure, such that larger numbers indicate greater similarities. Specifically, we calculated age_similarity = 1 – (age_difference/max(age_difference). To control for similarities in self-reported gender, we created an indicator variable in which 0 signifies different genders and 1 signifies the same gender. To control for similarities in ethnicity, we created an indicator variable for each race/ethnicity category (Asian, Black/African, Hispanic/Latinx, Native American, Pacific Islander, and Caucasian/White) in which 0 signifies a different self-reported race/ethnicity and 1 signifies the same self-reported race/ethnicity. The participants were able to self-report as many races/ethnicities as they desired. For each dyad, we created an overall indicator variable for race/ethnicity in which 0 signifies no shared race/ethnicity and 1 signifies at least one shared race/ethnicity. That is, if the two individuals in a dyad self-reported even one common race/ethnicity, we coded them as having a shared race/ethnicity. To control for similarities in home country, we created an additional indicator variable in which 0 signifies different home countries and 1 signifies the same home country. If either individual in a dyad nominated the other as a friend in the social-network survey, we coded the dyad as signifying an undirected friendship.

**Supplementary table: Descriptions of stimuli**

Table S1. Descriptions of stimuli

|  | Run # | Video | Content |
| --- | --- | --- | --- |
| 1 | 1 | An Astronaut’s View of Earth | An astronaut discusses viewing Earth from space and, in particular, witnessing the effects of climate change from space. He then urges viewers to mobilize to address this issue. |
| 2 | 1 | All I Want | A sentimental music video depicting a social outcast with a facial deformity who is seeking companionship. |
| 3 | 1 | Scientific demonstration | An astronaut at the International Space Station demonstrates and explains what happens when one wrings out a waterlogged washcloth in space. |
| 4 | 1 | Food Inc. | An excerpt from a documentary discussing how the fast-food industry influences food production and farming practices in the United States. |
| 5 | 2 | We Can Be Heroes | An excerpt from a mockumentary-style series in which a man discusses why he nominated himself for the title of Australian of the Year. |
| 6 | 2 | Ban College Football | Journalists and athletes debate whether football should be banned as a college sport. |
| 7 | 2 | Soccer match | Highlights from a soccer match. |
| 8 | 2 | Ew! | A comedy skit in which grown men play teenage girls disgusted by the things around them. |
| 9 | 2 | Life’s Too Short | An example of a ‘cringe comedy’ in which a dramatic actor is depicted unsuccessfully trying his hand at improvisational comedy. |
| 10 | 2 | America’s Funniest Home Videos | A series of homemade video clips that depict examples of unintentional physical comedy arising from accidents. |
| 11 | 3 | Zima Blue | A philosophical, animated short set in a futuristic world. |
| 12 | 3 | Nathan For You | An episode from a ‘docu-reality’ comedy in which the host convinces people, who are not always in on the joke, to engage in a variety of strange behaviors. |
| 13 | 4 | College Party | An excerpt from a film depicting a party scene in which a bashful college student is pressured to drink alcohol. |
| 14 | 4 | Eighth Grade | Two excerpts from a film. They depict (a) a young teenager who video blogs about her mental-health issues and (b) an awkward scene between two teenagers on a dinner date. |

Note: The video files for the stimuli that we used in this study are available at <https://gitlab.com/anon_authors/dorm_study_stimuli>

**Supplementary figure: Distribution of loneliness**

**
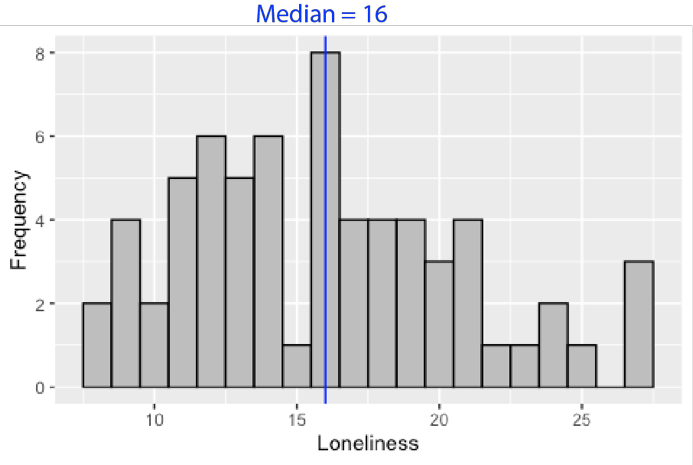
**

**Fig. S1.** Distribution of loneliness scores of the present study’s participants. We measured loneliness using the UCLA Loneliness Scale (ULS-8) *(36)*.

**Supplementary figure: Results associating neural similarity with maximum loneliness in dyads**


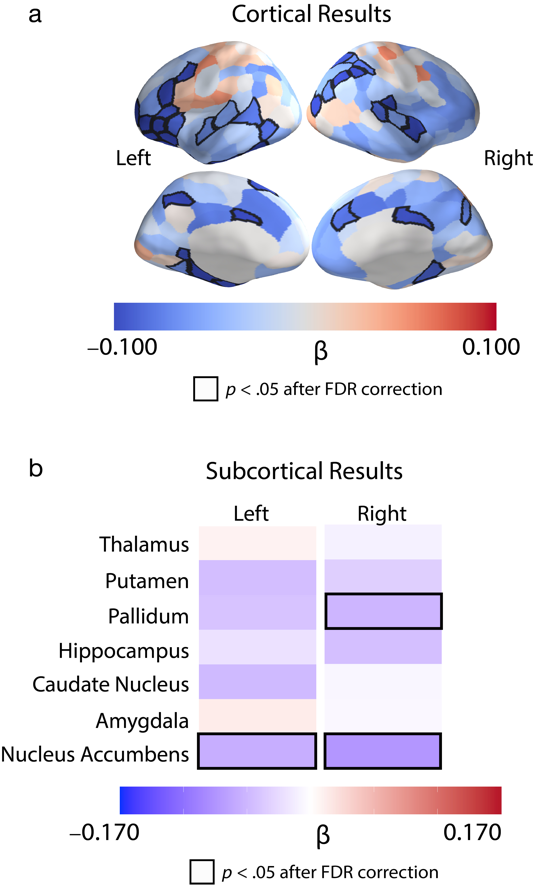


**Fig. S2.** Associating neural similarity with the maximum loneliness values in dyads. **(a)** We observed a negative association between ISCs and the maximum loneliness value. ISCs in brain regions (including the ventrolateral prefrontal cortex, dorsolateral prefrontal cortex, precuneus, posterior cingulate cortex, superior temporal cortex, inferior parietal lobule, and superior parietal lobule) that are associated with social cognition and shared understanding of events were associated with lower values of maximum loneliness. **(b)** There was a negative association between ISCs and maximum loneliness in the left and right nucleus accumbens and the right pallidum. The labels “Left” and “Right” refer to the hemispheres of the brain regions that are listed in the left panel. The quantity β is the standardized regression coefficient. Regions with significant associations between the maximum loneliness and ISC are outlined in black (using an FDR-corrected significance threshold of *p* < .05).

**Supplementary results: Supplementary tables**

In this section, we give tables of numerical values that are associated with various figures in the main manuscript.

Table S2. Results linking loneliness and neural responses: Cortical results

(corresponding to Fig. 2a of the main manuscript)

Contrast: ISC_{lonely, lonely}_ > ISC_{nonlonely, nonlonely}_

| Hemisphere | Network | Component Name^1^ | Parcel Number | β | SE | *p* value  (corrected) |
| --- | --- | --- | --- | --- | --- | --- |
| Left | Control | Parietal | 1 | −0.736 | 0.209 | 0.000 |
| Right | Dorsal Attention | Posterior | 4 | −0.783 | 0.250 | 0.001 |
| Right | Salience / Ventral Attention | Frontal Operculum | 2 | −0.713 | 0.232 | 0.001 |
| Left | Default | Parahippocampal Cortex | 1 | −0.758 | 0.249 | 0.001 |
| Right | Control | Parietal | 1 | −0.649 | 0.219 | 0.001 |
| Right | Control | Parietal | 2 | −0.675 | 0.228 | 0.001 |
| Right | Control | Lateral Prefrontal Cortex | 3 | −0.598 | 0.204 | 0.001 |
| Left | Somatomotor | Somatomotor | 2 | −0.897 | 0.308 | 0.001 |
| Right | Control | Lateral Prefrontal Cortex | 6 | −0.547 | 0.190 | 0.001 |
| Right | Control | Temporal | 1 | −0.678 | 0.244 | 0.002 |
| Right | Visual | Visual | 2 | −0.786 | 0.287 | 0.002 |
| Left | Dorsal Attention | Posterior | 7 | −0.774 | 0.283 | 0.002 |
| Right | Control | Precuneus | 1 | −0.713 | 0.263 | 0.002 |
| Left | Control | Lateral Prefrontal Cortex | 1 | −0.575 | 0.214 | 0.002 |
| Left | Default | Temporal | 5 | −0.792 | 0.298 | 0.003 |
| Right | Somatomotor | Somatomotor | 4 | −0.582 | 0.223 | 0.003 |
| Left | Control | Parietal | 2 | −0.643 | 0.249 | 0.003 |
| Left | Control | Lateral Prefrontal Cortex | 3 | −0.470 | 0.183 | 0.003 |
| Left | Default | Prefrontal Cortex | 12 | −0.520 | 0.203 | 0.003 |
| Left | Default | Temporal | 8 | −0.620 | 0.241 | 0.003 |
| Left | Somatomotor | Somatomotor | 1 | −0.768 | 0.300 | 0.003 |
| Left | Salience / Ventral Attention | Frontal Operculum | 2 | −0.534 | 0.209 | 0.003 |
| Left | Default | Prefrontal Cortex | 1 | −0.537 | 0.211 | 0.003 |
| Left | Control | Parietal | 3 | −0.640 | 0.259 | 0.005 |
| Left | Control | Lateral Prefrontal Cortex | 5 | −0.660 | 0.270 | 0.005 |
| Right | Dorsal Attention | Posterior | 8 | −0.692 | 0.283 | 0.005 |
| Right | Salience / Ventral Attention | Frontal Operculum | 1 | −0.459 | 0.188 | 0.005 |
| Left | Salience / Ventral Attention | Parietal Operculum | 3 | −0.539 | 0.222 | 0.005 |
| Left | Control | Lateral Prefrontal Cortex | 2 | −0.510 | 0.211 | 0.005 |
| Right | Salience / Ventral Attention | Frontal Operculum | 3 | −0.534 | 0.222 | 0.006 |
| Left | Limbic | Orbital Frontal Cortex | 1 | −0.405 | 0.170 | 0.006 |
| Left | Control | Lateral Prefrontal Cortex | 4 | −0.618 | 0.259 | 0.006 |
| Right | Visual | Visual | 11 | −0.624 | 0.264 | 0.006 |
| Left | Default | Posterior Cingulate Cortex | 3 | −0.452 | 0.195 | 0.007 |
| Right | Default | Posterior Cingulate Cortex | 1 | −0.628 | 0.274 | 0.008 |
| Right | Control | Medial Posterior Prefrontal Cortex | 1 | −0.525 | 0.230 | 0.008 |
| Left | Default | Prefrontal Cortex | 13 | −0.553 | 0.244 | 0.009 |
| Left | Default | Prefrontal Cortex | 10 | −0.470 | 0.213 | 0.011 |
| Right | Default | Parietal | 1 | −0.564 | 0.256 | 0.011 |
| Left | Control | Lateral Prefrontal Cortex | 6 | −0.535 | 0.244 | 0.011 |
| Left | Default | Prefrontal Cortex | 5 | −0.592 | 0.273 | 0.011 |
| Right | Dorsal Attention | Posterior | 3 | −0.539 | 0.250 | 0.012 |
| Right | Somatomotor | Somatomotor | 1 | −0.664 | 0.311 | 0.013 |
| Left | Control | Precuneus | 1 | −0.539 | 0.253 | 0.013 |
| Left | Default | Prefrontal Cortex | 3 | −0.523 | 0.246 | 0.013 |
| Left | Default | Prefrontal Cortex | 8 | −0.384 | 0.181 | 0.013 |
| Right | Default | Posterior Cingulate Cortex | 2 | −0.546 | 0.257 | 0.013 |
| Right | Default | Ventral Prefrontal Cortex | 1 | −0.509 | 0.241 | 0.014 |
| Right | Control | Medial Posterior Prefrontal Cortex | 3 | −0.379 | 0.181 | 0.015 |
| Right | Control | Ventral Prefrontal Cortex | 1 | −0.449 | 0.215 | 0.015 |
| Right | Visual | Visual | 15 | −0.634 | 0.303 | 0.015 |
| Left | Salience / Ventral Attention | Parietal Operculum | 1 | −0.548 | 0.267 | 0.016 |
| Right | Default | Medial Prefrontal Cortex | 7 | −0.439 | 0.216 | 0.017 |
| Right | Control | Parietal | 3 | −0.501 | 0.248 | 0.018 |
| Right | Default | Parietal | 3 | −0.486 | 0.241 | 0.018 |
| Left | Default | Prefrontal Cortex | 11 | −0.433 | 0.216 | 0.018 |
| Left | Default | Posterior Cingulate Cortex | 2 | −0.524 | 0.262 | 0.019 |
| Left | Default | Posterior Cingulate Cortex | 1 | −0.515 | 0.259 | 0.019 |
| Left | Default | Posterior Cingulate Cortex | 4 | −0.510 | 0.258 | 0.020 |
| Right | Dorsal Attention | Frontal Eye Fields | 2 | −0.481 | 0.244 | 0.020 |
| Left | Default | Prefrontal Cortex | 9 | −0.477 | 0.243 | 0.021 |
| Right | Dorsal Attention | Posterior | 10 | −0.490 | 0.251 | 0.022 |
| Left | Default | Temporal | 3 | −0.584 | 0.301 | 0.023 |
| Right | Somatomotor | Somatomotor | 2 | −0.595 | 0.310 | 0.024 |
| Right | Visual | Visual | 1 | −0.513 | 0.273 | 0.028 |
| Left | Dorsal Attention | Posterior | 10 | −0.504 | 0.271 | 0.029 |
| Left | Salience / Ventral Attention | Lateral Prefrontal Cortex | 1 | −0.377 | 0.204 | 0.031 |
| Left | Visual | Visual | 1 | −0.545 | 0.297 | 0.032 |
| Left | Dorsal Attention | Frontal Eye Fields | 2 | −0.476 | 0.261 | 0.032 |
| Right | Dorsal Attention | Posterior | 7 | −0.504 | 0.279 | 0.035 |
| Right | Control | Lateral Prefrontal Cortex | 7 | −0.423 | 0.235 | 0.035 |
| Left | Dorsal Attention | Posterior | 4 | −0.483 | 0.279 | 0.043 |
| Right | Control | Medial Posterior Prefrontal Cortex | 4 | −0.423 | 0.245 | 0.044 |
| Left | Dorsal Attention | Posterior | 1 | −0.455 | 0.269 | 0.048 |

^1^Component names reflect the labels that were provided by Yeo et al. (2011). These labels split the seven networks into spatially connected components. We provide the component names and intra-component parcel numbers to aid in the identification of the parcels.

Note: The component names and the parcel numbers (within components) correspond to those that accompany the Schaefer et al., (2018) parcellation. Each row represents a single parcel in the brain. Component names reflect the component labels. More information about each parcel is available at their GitHub page (<https://github.com/ThomasYeoLab/CBIG/tree/master/stable_projects/brain_parcellation/Schaefer2018_LocalGlobal>). The quantity β is the standardized regression coefficient and the quantity SE is the standard error.

Table S3. Results linking loneliness and neural responses: Cortical results

(corresponding to Fig. 2a of the main manuscript)

Contrast: ISC_{lonely, lonely}_ > ISC_{nonlonely, lonely}_

| Hemisphere | Network | Component Name^1^ | Parcel Number | β | SE | *p* value  (corrected) |
| --- | --- | --- | --- | --- | --- | --- |
| Left | Control | Parietal | 1 | −0.343 | 0.109 | 0.001 |
| Right | Dorsal Attention | Posterior | 4 | −0.370 | 0.128 | 0.001 |
| Right | Salience / Ventral Attention | Frontal Operculum | 2 | −0.335 | 0.120 | 0.002 |
| Right | Control | Lateral Prefrontal Cortex | 6 | −0.281 | 0.101 | 0.002 |
| Left | Dorsal Attention | Posterior | 7 | −0.391 | 0.143 | 0.002 |
| Right | Control | Lateral Prefrontal Cortex | 3 | −0.286 | 0.107 | 0.002 |
| Left | Default | Prefrontal Cortex | 12 | −0.282 | 0.107 | 0.003 |
| Left | Salience / Ventral Attention | Parietal Operculum | 3 | −0.305 | 0.115 | 0.003 |
| Right | Control | Parietal | 1 | −0.298 | 0.114 | 0.003 |
| Left | Somatomotor | Somatomotor | 2 | −0.401 | 0.155 | 0.003 |
| Right | Control | Precuneus | 1 | −0.328 | 0.134 | 0.005 |
| Right | Control | Temporal | 1 | −0.306 | 0.125 | 0.005 |
| Right | Default | Posterior Cingulate Cortex | 1 | −0.338 | 0.139 | 0.005 |
| Right | Control | Parietal | 2 | −0.285 | 0.118 | 0.005 |
| Left | Default | Prefrontal Cortex | 10 | −0.264 | 0.111 | 0.006 |
| Right | Visual | Visual | 2 | −0.345 | 0.145 | 0.006 |
| Left | Default | Temporal | 8 | −0.293 | 0.124 | 0.006 |
| Left | Control | Parietal | 3 | −0.311 | 0.132 | 0.007 |
| Left | Default | Posterior Cingulate Cortex | 2 | −0.311 | 0.134 | 0.007 |
| Right | Dorsal Attention | Posterior | 3 | −0.298 | 0.128 | 0.007 |
| Left | Default | Parahippocampal Cortex | 1 | −0.293 | 0.127 | 0.008 |
| Left | Control | Lateral Prefrontal Cortex | 1 | −0.253 | 0.112 | 0.009 |
| Left | Default | Temporal | 5 | −0.338 | 0.150 | 0.009 |
| Left | Salience / Ventral Attention | Frontal Operculum | 2 | −0.246 | 0.109 | 0.009 |
| Right | Somatomotor | Somatomotor | 4 | −0.259 | 0.116 | 0.009 |
| Left | Default | Prefrontal Cortex | 1 | −0.243 | 0.110 | 0.011 |
| Right | Salience / Ventral Attention | Frontal Operculum | 3 | −0.255 | 0.115 | 0.011 |
| Left | Control | Lateral Prefrontal Cortex | 4 | −0.290 | 0.132 | 0.011 |
| Left | Control | Lateral Prefrontal Cortex | 5 | −0.299 | 0.137 | 0.011 |
| Right | Salience / Ventral Attention | Frontal Operculum | 1 | −0.219 | 0.100 | 0.011 |
| Left | Control | Parietal | 2 | −0.278 | 0.127 | 0.011 |
| Left | Default | Prefrontal Cortex | 13 | −0.271 | 0.125 | 0.012 |
| Right | Default | Posterior Cingulate Cortex | 2 | −0.283 | 0.131 | 0.012 |
| Left | Somatomotor | Somatomotor | 1 | −0.324 | 0.151 | 0.013 |
| Right | Dorsal Attention | Frontal Eye Fields | 2 | −0.265 | 0.125 | 0.014 |
| Left | Default | Posterior Cingulate Cortex | 1 | −0.276 | 0.132 | 0.015 |
| Left | Default | Posterior Cingulate Cortex | 3 | −0.216 | 0.103 | 0.015 |
| Right | Control | Ventral Prefrontal Cortex | 1 | −0.234 | 0.112 | 0.015 |
| Left | Control | Precuneus | 1 | −0.270 | 0.130 | 0.015 |
| Left | Default | Prefrontal Cortex | 9 | −0.257 | 0.125 | 0.015 |
| Right | Default | Parietal | 1 | −0.269 | 0.131 | 0.016 |
| Left | Default | Prefrontal Cortex | 11 | −0.228 | 0.113 | 0.018 |
| Left | Salience / Ventral Attention | Parietal Operculum | 1 | −0.275 | 0.136 | 0.018 |
| Left | Dorsal Attention | Posterior | 10 | −0.279 | 0.138 | 0.018 |
| Right | Visual | Visual | 11 | −0.272 | 0.135 | 0.018 |
| Right | Dorsal Attention | Posterior | 8 | −0.286 | 0.144 | 0.019 |
| Left | Default | Prefrontal Cortex | 3 | −0.248 | 0.126 | 0.021 |
| Right | Control | Medial Posterior Prefrontal Cortex | 1 | −0.233 | 0.119 | 0.021 |
| Right | Visual | Visual | 15 | −0.298 | 0.153 | 0.022 |
| Right | Somatomotor | Somatomotor | 1 | −0.300 | 0.156 | 0.024 |
| Left | Control | Lateral Prefrontal Cortex | 2 | −0.211 | 0.110 | 0.024 |
| Right | Dorsal Attention | Posterior | 10 | −0.245 | 0.129 | 0.026 |
| Right | Default | Medial Prefrontal Cortex | 7 | −0.211 | 0.113 | 0.028 |
| Left | Dorsal Attention | Posterior | 4 | −0.262 | 0.142 | 0.030 |
| Right | Default | Parietal | 3 | −0.228 | 0.124 | 0.032 |
| Right | Dorsal Attention | Posterior | 7 | −0.257 | 0.142 | 0.033 |
| Left | Default | Posterior Cingulate Cortex | 4 | −0.237 | 0.132 | 0.035 |
| Left | Default | Prefrontal Cortex | 5 | −0.249 | 0.139 | 0.035 |
| Right | Dorsal Attention | Posterior | 6 | −0.247 | 0.137 | 0.035 |
| Right | Default | Medial Prefrontal Cortex | 6 | −0.211 | 0.119 | 0.038 |
| Left | Dorsal Attention | Posterior | 9 | −0.241 | 0.138 | 0.041 |
| Right | Default | Ventral Prefrontal Cortex | 1 | −0.216 | 0.124 | 0.042 |
| Right | Control | Parietal | 3 | −0.220 | 0.127 | 0.043 |
| Left | Control | Temporal | 1 | −0.223 | 0.129 | 0.043 |
| Left | Default | Temporal | 3 | −0.263 | 0.152 | 0.043 |
| Left | Dorsal Attention | Frontal Eye Fields | 2 | −0.229 | 0.133 | 0.045 |
| Left | Salience / Ventral Attention | Medial | 3 | −0.198 | 0.115 | 0.045 |
| Left | Visual | Visual | 1 | −0.255 | 0.150 | 0.048 |

^1^Component names reflect the labels that were provided by Yeo et al. (2011). These labels split the seven networks into spatially connected components. We provide the component names and intra-component parcel numbers to aid in the identification of the parcels.

Note: The component names and the parcel numbers (within components) correspond to those that accompany the Schaefer et al., (2018) parcellation. Each row represents a single parcel in the brain. Component names reflect the component labels. More information about each parcel is available at their GitHub page (<https://github.com/ThomasYeoLab/CBIG/tree/master/stable_projects/brain_parcellation/Schaefer2018_LocalGlobal>). The quantity β is the standardized regression coefficient and the quantity SE is the standard error.

Table S4. Results linking loneliness and neural responses: Cortical results

(corresponding to Fig. 2a of the main manuscript)

Contrast: ISC_{nonlonely, lonely}_ > ISC_{nonlonely, nonlonely}_

| Hemisphere | Network | Component Name^1^ | Parcel Number | β | SE | *p* value  (corrected) |
| --- | --- | --- | --- | --- | --- | --- |
| Left | Control | Parietal | 1 | −0.393 | 0.111 | 0.000 |
| Left | Default | Parahippocampal Cortex | 1 | −0.465 | 0.128 | 0.000 |
| Right | Control | Parietal | 2 | −0.390 | 0.119 | 0.001 |
| Left | Control | Lateral Prefrontal Cortex | 3 | −0.314 | 0.100 | 0.001 |
| Left | Somatomotor | Somatomotor | 2 | −0.495 | 0.155 | 0.001 |
| Right | Dorsal Attention | Posterior | 4 | −0.414 | 0.129 | 0.001 |
| Right | Salience / Ventral Attention | Frontal Operculum | 2 | −0.377 | 0.121 | 0.001 |
| Right | Control | Parietal | 1 | −0.351 | 0.115 | 0.001 |
| Right | Visual | Visual | 2 | −0.442 | 0.146 | 0.001 |
| Left | Default | Temporal | 5 | −0.455 | 0.151 | 0.001 |
| Right | Control | Temporal | 1 | −0.372 | 0.126 | 0.001 |
| Left | Somatomotor | Somatomotor | 1 | −0.444 | 0.152 | 0.001 |
| Left | Control | Lateral Prefrontal Cortex | 6 | −0.368 | 0.127 | 0.001 |
| Right | Control | Lateral Prefrontal Cortex | 3 | −0.311 | 0.108 | 0.001 |
| Left | Control | Lateral Prefrontal Cortex | 1 | −0.323 | 0.113 | 0.001 |
| Right | Control | Precuneus | 1 | −0.385 | 0.135 | 0.001 |
| Left | Control | Parietal | 2 | −0.365 | 0.128 | 0.001 |
| Right | Dorsal Attention | Posterior | 8 | −0.407 | 0.144 | 0.001 |
| Right | Somatomotor | Somatomotor | 4 | −0.322 | 0.117 | 0.002 |
| Left | Limbic | Orbital Frontal Cortex | 1 | −0.254 | 0.094 | 0.002 |
| Left | Control | Lateral Prefrontal Cortex | 2 | −0.299 | 0.112 | 0.002 |
| Left | Dorsal Attention | Posterior | 7 | −0.383 | 0.144 | 0.003 |
| Left | Default | Prefrontal Cortex | 1 | −0.294 | 0.112 | 0.003 |
| Left | Control | Lateral Prefrontal Cortex | 5 | −0.360 | 0.138 | 0.003 |
| Left | Default | Temporal | 8 | −0.327 | 0.125 | 0.003 |
| Left | Salience / Ventral Attention | Frontal Operculum | 2 | −0.288 | 0.111 | 0.003 |
| Right | Visual | Visual | 11 | −0.352 | 0.135 | 0.003 |
| Right | Control | Lateral Prefrontal Cortex | 6 | −0.265 | 0.102 | 0.003 |
| Left | Default | Prefrontal Cortex | 8 | −0.253 | 0.099 | 0.003 |
| Right | Control | Medial Posterior Prefrontal Cortex | 3 | −0.248 | 0.099 | 0.004 |
| Left | Control | Parietal | 3 | −0.330 | 0.133 | 0.005 |
| Left | Control | Lateral Prefrontal Cortex | 4 | −0.328 | 0.133 | 0.005 |
| Left | Default | Prefrontal Cortex | 5 | −0.343 | 0.139 | 0.005 |
| Right | Somatomotor | Somatomotor | 2 | −0.386 | 0.156 | 0.005 |
| Right | Control | Medial Posterior Prefrontal Cortex | 1 | −0.292 | 0.120 | 0.005 |
| Right | Salience / Ventral Attention | Frontal Operculum | 3 | −0.280 | 0.117 | 0.006 |
| Right | Salience / Ventral Attention | Frontal Operculum | 1 | −0.241 | 0.102 | 0.006 |
| Right | Default | Ventral Prefrontal Cortex | 1 | −0.293 | 0.125 | 0.007 |
| Right | Somatomotor | Somatomotor | 1 | −0.363 | 0.157 | 0.007 |
| Left | Dorsal Attention | Posterior | 1 | −0.316 | 0.138 | 0.008 |
| Left | Default | Posterior Cingulate Cortex | 3 | −0.236 | 0.105 | 0.009 |
| Right | Default | Parietal | 1 | −0.296 | 0.132 | 0.009 |
| Left | Default | Prefrontal Cortex | 13 | −0.282 | 0.126 | 0.010 |
| Left | Default | Prefrontal Cortex | 12 | −0.238 | 0.108 | 0.011 |
| Right | Control | Parietal | 3 | −0.281 | 0.128 | 0.011 |
| Right | Visual | Visual | 15 | −0.336 | 0.153 | 0.011 |
| Left | Default | Prefrontal Cortex | 3 | −0.274 | 0.127 | 0.012 |
| Right | Visual | Visual | 1 | −0.296 | 0.140 | 0.013 |
| Left | Default | Temporal | 3 | −0.321 | 0.152 | 0.014 |
| Left | Control | Cingulate | 2 | −0.215 | 0.103 | 0.015 |
| Right | Control | Medial Posterior Prefrontal Cortex | 4 | −0.265 | 0.127 | 0.015 |
| Right | Default | Posterior Cingulate Cortex | 1 | −0.290 | 0.140 | 0.015 |
| Left | Control | Precuneus | 1 | −0.270 | 0.131 | 0.015 |
| Right | Default | Parietal | 3 | −0.258 | 0.125 | 0.015 |
| Left | Default | Posterior Cingulate Cortex | 4 | −0.273 | 0.133 | 0.016 |
| Left | Salience / Ventral Attention | Parietal Operculum | 3 | −0.234 | 0.116 | 0.018 |
| Right | Default | Medial Prefrontal Cortex | 7 | −0.228 | 0.114 | 0.019 |
| Left | Salience / Ventral Attention | Parietal Operculum | 1 | −0.272 | 0.137 | 0.019 |
| Right | Default | Posterior Cingulate Cortex | 2 | −0.263 | 0.132 | 0.019 |
| Left | Visual | Visual | 1 | −0.290 | 0.151 | 0.024 |
| Right | Control | Ventral Prefrontal Cortex | 1 | −0.215 | 0.114 | 0.027 |
| Right | Dorsal Attention | Posterior | 10 | −0.245 | 0.130 | 0.027 |
| Right | Dorsal Attention | Posterior | 3 | −0.241 | 0.129 | 0.029 |
| Left | Dorsal Attention | Frontal Eye Fields | 2 | −0.248 | 0.134 | 0.030 |
| Left | Default | Prefrontal Cortex | 10 | −0.206 | 0.112 | 0.032 |
| Left | Salience / Ventral Attention | Lateral Prefrontal Cortex | 1 | −0.197 | 0.109 | 0.035 |
| Left | Default | Prefrontal Cortex | 11 | −0.205 | 0.114 | 0.035 |
| Right | Control | Lateral Prefrontal Cortex | 7 | −0.221 | 0.123 | 0.035 |
| Left | Default | Posterior Cingulate Cortex | 1 | −0.239 | 0.133 | 0.035 |
| Left | Dorsal Attention | Posterior | 2 | −0.203 | 0.113 | 0.035 |
| Left | Default | Prefrontal Cortex | 9 | −0.219 | 0.126 | 0.042 |
| Right | Dorsal Attention | Posterior | 7 | −0.247 | 0.142 | 0.043 |
| Right | Dorsal Attention | Frontal Eye Fields | 2 | −0.215 | 0.126 | 0.047 |

^1^Component names reflect the labels that were provided by Yeo et al. (2011). These labels split the seven networks into spatially connected components. We provide the component names and intra-component parcel numbers to aid in the identification of the parcels.

Note: The component names and the parcel numbers (within components) correspond to those that accompany the Schaefer et al., (2018) parcellation. Each row represents a single parcel in the brain. Component names reflect the component labels. More information about each parcel is available at their GitHub page (<https://github.com/ThomasYeoLab/CBIG/tree/master/stable_projects/brain_parcellation/Schaefer2018_LocalGlobal>). The quantity β is the standardized regression coefficient and the quantity SE is the standard error.

Table S5. Results linking loneliness and neural responses: Subcortical results

(corresponding to Fig. 2b of the main manuscript)

Contrast: ISC_{lonely, lonely}_ > ISC_{nonlonely, nonlonely}_

| Hemisphere | Brain Region | β | SE | *p* value (corrected) |
| --- | --- | --- | --- | --- |
| Left | Nucleus Accumbens | −0.259 | 0.110 | 0.007 |
| Right | Pallidum | −0.154 | 0.083 | 0.029 |
| Left | Caudate Nucleus | −0.289 | 0.160 | 0.035 |

Table S6. Results linking loneliness and neural responses: Subcortical results

(corresponding to Fig. 2b of the main manuscript)

Contrast: ISC_{nonlonely, lonely}_ > ISC_{nonlonely, nonlonely}_

| Hemisphere | Brain Region | β | SE | *p* value (corrected) |
| --- | --- | --- | --- | --- |
| Left | Caudate Nucleus | −0.203 | 0.090 | 0.009 |
| Left | Nucleus Accumbens | −0.153 | 0.071 | 0.012 |
| Right | Pallidum | −0.128 | 0.061 | 0.015 |
| Right | Nucleus Accumbens | −0.128 | 0.068 | 0.027 |

Table S7. Results linking loneliness and neural responses, controlling for objective social disconnection, demographic similarities, and friendships between participants: Cortical results

(corresponding to Fig. 4a of the main manuscript)

Contrast: ISC_{lonely, lonely}_ > ISC_{nonlonely, nonlonely}_

| Hemisphere | Network | Component Name^1^ | Parcel Number | β | SE | *p* value  (corrected) |
| --- | --- | --- | --- | --- | --- | --- |
| Left | Control | Parietal | 1 | −0.684 | 0.215 | 0.002 |
| Right | Salience / Ventral Attention | Frontal Operculum | 2 | −0.701 | 0.241 | 0.003 |
| Left | Somatomotor | Somatomotor | 2 | −0.867 | 0.320 | 0.007 |
| Right | Control | Lateral Prefrontal Cortex | 3 | −0.576 | 0.218 | 0.007 |
| Right | Visual | Visual | 2 | −0.785 | 0.298 | 0.007 |
| Right | Dorsal Attention | Posterior | 4 | −0.663 | 0.257 | 0.008 |
| Right | Control | Lateral Prefrontal Cortex | 6 | −0.505 | 0.200 | 0.010 |
| Left | Control | Lateral Prefrontal Cortex | 1 | −0.568 | 0.226 | 0.010 |
| Right | Control | Parietal | 2 | −0.602 | 0.240 | 0.010 |
| Left | Control | Parietal | 2 | −0.619 | 0.254 | 0.011 |
| Left | Default | Temporal | 5 | −0.762 | 0.309 | 0.011 |
| Right | Control | Parietal | 1 | −0.577 | 0.236 | 0.011 |
| Right | Control | Temporal | 1 | −0.632 | 0.260 | 0.011 |
| Left | Default | Parahippocampal Cortex | 1 | −0.611 | 0.253 | 0.011 |
| Right | Somatomotor | Somatomotor | 4 | −0.541 | 0.230 | 0.014 |
| Left | Dorsal Attention | Posterior | 7 | −0.691 | 0.294 | 0.014 |
| Left | Control | Lateral Prefrontal Cortex | 5 | −0.640 | 0.276 | 0.014 |
| Left | Somatomotor | Somatomotor | 1 | −0.730 | 0.314 | 0.014 |
| Left | Limbic | Orbital Frontal Cortex | 1 | −0.396 | 0.173 | 0.016 |
| Left | Control | Parietal | 3 | −0.598 | 0.264 | 0.016 |
| Left | Control | Lateral Prefrontal Cortex | 3 | −0.436 | 0.192 | 0.016 |
| Left | Control | Lateral Prefrontal Cortex | 4 | −0.578 | 0.263 | 0.019 |
| Right | Salience / Ventral Attention | Frontal Operculum | 1 | −0.415 | 0.193 | 0.021 |
| Left | Default | Prefrontal Cortex | 1 | −0.442 | 0.210 | 0.025 |
| Left | Salience / Ventral Attention | Frontal Operculum | 2 | −0.435 | 0.208 | 0.025 |
| Right | Default | Parietal | 1 | −0.527 | 0.252 | 0.025 |
| Right | Dorsal Attention | Posterior | 3 | −0.547 | 0.261 | 0.025 |
| Left | Control | Lateral Prefrontal Cortex | 2 | −0.444 | 0.215 | 0.028 |
| Left | Default | Prefrontal Cortex | 12 | −0.432 | 0.211 | 0.029 |
| Right | Default | Ventral Prefrontal Cortex | 1 | −0.508 | 0.249 | 0.029 |
| Left | Default | Temporal | 8 | −0.502 | 0.247 | 0.029 |
| Right | Salience / Ventral Attention | Frontal Operculum | 3 | −0.462 | 0.228 | 0.030 |
| Left | Salience / Ventral Attention | Parietal Operculum | 3 | −0.476 | 0.237 | 0.031 |
| Left | Default | Posterior Cingulate Cortex | 3 | −0.405 | 0.204 | 0.033 |
| Right | Visual | Visual | 11 | −0.526 | 0.266 | 0.033 |
| Left | Default | Prefrontal Cortex | 5 | −0.564 | 0.286 | 0.033 |
| Right | Somatomotor | Somatomotor | 1 | −0.632 | 0.320 | 0.033 |
| Left | Control | Lateral Prefrontal Cortex | 6 | −0.497 | 0.260 | 0.041 |

^1^Component names reflect the labels that were provided by Yeo et al. (2011). These labels split the seven networks into spatially connected components. We provide the component names and intra-component parcel numbers to aid in the identification of the parcels.

Note: The component names and the parcel numbers (within components) correspond to those that accompany the Schaefer et al., (2018) parcellation. Each row represents a single parcel in the brain. Component names reflect the component labels. More information about each parcel is available at their GitHub page (<https://github.com/ThomasYeoLab/CBIG/tree/master/stable_projects/brain_parcellation/Schaefer2018_LocalGlobal>). The quantity β is the standardized regression coefficient and the quantity SE is the standard error.

Table S8. Results linking loneliness and neural responses, controlling for objective social disconnection, demographic similarities, and friendships between participants: Subcortical results

(corresponding to Fig. 4b of the main manuscript)

Contrast: ISC_{lonely, lonely}_ > ISC_{nonlonely, nonlonely}_

| Hemisphere | Brain Region | β | SE | *p* value (corrected) |
| --- | --- | --- | --- | --- |
| Left | Nucleus Accumbens | −0.205 | 0.106 | 0.037 |

Table S9. Results linking loneliness and neural responses, controlling for objective social disconnection, demographic similarities, and friendships between participants: Cortical results

(corresponding to Fig. 4a of the main manuscript)

Contrast: ISC_{lonely, lonely}_ > ISC_{nonlonely, lonely}_

| Hemisphere | Network | Component Name^1^ | Parcel Number | β | SE | *p* value  (corrected) |
| --- | --- | --- | --- | --- | --- | --- |
| Left | Control | Parietal | 1 | −0.321 | 0.113 | 0.004 |
| Right | Salience / Ventral Attention | Frontal Operculum | 2 | −0.323 | 0.125 | 0.008 |
| Left | Dorsal Attention | Posterior | 7 | −0.357 | 0.149 | 0.012 |
| Right | Control | Lateral Prefrontal Cortex | 3 | −0.275 | 0.115 | 0.012 |
| Right | Dorsal Attention | Posterior | 4 | −0.317 | 0.132 | 0.012 |
| Left | Somatomotor | Somatomotor | 2 | −0.384 | 0.161 | 0.012 |
| Right | Control | Lateral Prefrontal Cortex | 6 | −0.244 | 0.107 | 0.016 |
| Right | Dorsal Attention | Posterior | 3 | −0.304 | 0.134 | 0.016 |
| Right | Visual | Visual | 2 | −0.339 | 0.151 | 0.017 |
| Left | Control | Parietal | 3 | −0.301 | 0.135 | 0.017 |
| Left | Salience / Ventral Attention | Parietal Operculum | 3 | −0.271 | 0.123 | 0.019 |
| Right | Control | Parietal | 1 | −0.270 | 0.123 | 0.019 |
| Left | Default | Prefrontal Cortex | 12 | −0.241 | 0.112 | 0.021 |
| Right | Control | Temporal | 1 | −0.288 | 0.133 | 0.021 |
| Left | Control | Lateral Prefrontal Cortex | 1 | −0.250 | 0.118 | 0.025 |
| Left | Control | Parietal | 2 | −0.274 | 0.131 | 0.025 |
| Left | Control | Lateral Prefrontal Cortex | 5 | −0.294 | 0.141 | 0.025 |
| Right | Somatomotor | Somatomotor | 4 | −0.246 | 0.120 | 0.029 |
| Right | Dorsal Attention | Frontal Eye Fields | 2 | −0.266 | 0.131 | 0.029 |
| Left | Control | Lateral Prefrontal Cortex | 4 | −0.274 | 0.135 | 0.029 |
| Right | Control | Parietal | 2 | −0.250 | 0.125 | 0.031 |
| Left | Default | Prefrontal Cortex | 10 | −0.228 | 0.115 | 0.033 |
| Right | Default | Parietal | 1 | −0.255 | 0.130 | 0.035 |
| Left | Default | Temporal | 5 | −0.305 | 0.156 | 0.035 |
| Left | Default | Temporal | 8 | −0.249 | 0.128 | 0.035 |
| Left | Somatomotor | Somatomotor | 1 | −0.310 | 0.159 | 0.035 |
| Right | Salience / Ventral Attention | Frontal Operculum | 3 | −0.231 | 0.119 | 0.037 |

^1^Component names reflect the labels that were provided by Yeo et al. (2011). These labels split the seven networks into spatially connected components. We provide the component names and intra-component parcel numbers to aid in the identification of the parcels.

Note: The component names and the parcel numbers (within components) correspond to those that accompany the Schaefer et al., (2018) parcellation. Each row represents a single parcel in the brain. Component names reflect the component labels. More information about each parcel is available at their GitHub page (<https://github.com/ThomasYeoLab/CBIG/tree/master/stable_projects/brain_parcellation/Schaefer2018_LocalGlobal>). The quantity β is the standardized regression coefficient and the quantity SE is the standard error.

Table S10. Results linking loneliness and neural responses, controlling for objective social disconnection, demographic similarities, and friendships between participants: Cortical results

(corresponding to Fig. 4a of the main manuscript)

Contrast: ISC_{nonlonely, lonely}_ > ISC_{nonlonely, nonlonely}_

| Hemisphere | Network | Component Name^1^ | Parcel Number | β | SE | *p* value  (corrected) |
| --- | --- | --- | --- | --- | --- | --- |
| Left | Control | Parietal | 1 | −0.363 | 0.113 | 0.002 |
| Left | Default | Parahippocampal Cortex | 1 | −0.396 | 0.130 | 0.003 |
| Left | Somatomotor | Somatomotor | 2 | −0.484 | 0.161 | 0.003 |
| Right | Salience / Ventral Attention | Frontal Operculum | 2 | −0.378 | 0.125 | 0.003 |
| Left | Default | Temporal | 5 | −0.456 | 0.156 | 0.003 |
| Right | Visual | Visual | 2 | −0.447 | 0.151 | 0.003 |
| Left | Control | Lateral Prefrontal Cortex | 3 | −0.296 | 0.104 | 0.004 |
| Right | Control | Parietal | 2 | −0.352 | 0.125 | 0.004 |
| Left | Control | Parietal | 2 | −0.345 | 0.131 | 0.007 |
| Left | Control | Lateral Prefrontal Cortex | 1 | −0.318 | 0.119 | 0.007 |
| Left | Limbic | Orbital Frontal Cortex | 1 | −0.254 | 0.096 | 0.007 |
| Left | Somatomotor | Somatomotor | 1 | −0.420 | 0.159 | 0.007 |
| Right | Control | Lateral Prefrontal Cortex | 3 | −0.301 | 0.115 | 0.007 |
| Right | Dorsal Attention | Posterior | 4 | −0.345 | 0.132 | 0.007 |
| Right | Control | Temporal | 1 | −0.344 | 0.134 | 0.008 |
| Right | Control | Parietal | 1 | −0.307 | 0.123 | 0.010 |
| Left | Control | Lateral Prefrontal Cortex | 5 | −0.346 | 0.141 | 0.011 |
| Left | Control | Lateral Prefrontal Cortex | 6 | −0.326 | 0.134 | 0.011 |
| Right | Control | Lateral Prefrontal Cortex | 6 | −0.260 | 0.107 | 0.011 |
| Right | Somatomotor | Somatomotor | 4 | −0.295 | 0.120 | 0.011 |
| Left | Default | Prefrontal Cortex | 1 | −0.261 | 0.112 | 0.014 |
| Left | Default | Prefrontal Cortex | 5 | −0.339 | 0.146 | 0.014 |
| Left | Default | Prefrontal Cortex | 8 | −0.229 | 0.099 | 0.014 |
| Right | Somatomotor | Somatomotor | 2 | −0.375 | 0.162 | 0.014 |
| Left | Control | Lateral Prefrontal Cortex | 4 | −0.304 | 0.135 | 0.017 |
| Left | Dorsal Attention | Posterior | 7 | −0.334 | 0.149 | 0.017 |
| Left | Salience / Ventral Attention | Frontal Operculum | 2 | −0.248 | 0.111 | 0.017 |
| Right | Default | Ventral Prefrontal Cortex | 1 | −0.288 | 0.129 | 0.017 |
| Right | Salience / Ventral Attention | Frontal Operculum | 1 | −0.233 | 0.104 | 0.017 |
| Right | Visual | Visual | 11 | −0.302 | 0.136 | 0.018 |
| Left | Control | Parietal | 3 | −0.297 | 0.136 | 0.019 |
| Right | Somatomotor | Somatomotor | 1 | −0.353 | 0.161 | 0.019 |
| Left | Control | Lateral Prefrontal Cortex | 2 | −0.248 | 0.114 | 0.019 |
| Right | Control | Medial Posterior Prefrontal Cortex | 3 | −0.213 | 0.101 | 0.025 |
| Right | Default | Parietal | 1 | −0.272 | 0.130 | 0.025 |
| Right | Control | Parietal | 3 | −0.271 | 0.131 | 0.027 |
| Right | Dorsal Attention | Posterior | 8 | −0.305 | 0.149 | 0.029 |
| Left | Default | Posterior Cingulate Cortex | 3 | −0.219 | 0.109 | 0.031 |
| Right | Control | Precuneus | 1 | −0.272 | 0.137 | 0.033 |
| Left | Default | Temporal | 8 | −0.253 | 0.128 | 0.033 |
| Right | Salience / Ventral Attention | Frontal Operculum | 3 | −0.231 | 0.120 | 0.038 |
| Right | Control | Medial Posterior Prefrontal Cortex | 1 | −0.230 | 0.121 | 0.042 |
| Right | Default | Medial Prefrontal Cortex | 7 | −0.222 | 0.119 | 0.047 |

^1^Component names reflect the labels that were provided by Yeo et al. (2011). These labels split the seven networks into spatially connected components. We provide the component names and intra-component parcel numbers to aid in the identification of the parcels.

Note: The component names and the parcel numbers (within components) correspond to those that accompany the Schaefer et al., (2018) parcellation. Each row represents a single parcel in the brain. Component names reflect the component labels. More information about each parcel is available at their GitHub page (<https://github.com/ThomasYeoLab/CBIG/tree/master/stable_projects/brain_parcellation/Schaefer2018_LocalGlobal>). The quantity β is the standardized regression coefficient and the quantity SE is the standard error.

Table S11. Results linking loneliness and neural responses, controlling for objective social disconnection, demographic similarities, and friendships between participants: Subcortical results

(corresponding to Fig. 4b of the main manuscript)

Contrast: ISC_{nonlonely, lonely}_ > ISC_{nonlonely, nonlonely}_

| Hemisphere | Brain Region | β | SE | *p* value (corrected) |
| --- | --- | --- | --- | --- |
| Right | Pallidum | −0.142 | 0.062 | 0.016 |
| Left | Caudate Nucleus | −0.195 | 0.093 | 0.025 |

**Supplementary results: Objective social disconnection**

**Objective social disconnection**. As we noted in the main manuscript, we used out-degree centrality as a measure of objective social disconnection. The out-degree centrality of the participants ranged from 0 to 23 (with mean = 4.73, median = 3, and *SD* = 5.153).

We split the participants into two groups based on a median split of their objective social-disconnection measure. We categorized participants into the low objective social-disconnection group if they had an out-degree that was larger than the median (specifically, if it was more than 3; there were *n*_low_ = 25 such people) and into the high objective social-disconnection group if they had an out-degree that was less than or equal to the median (specifically, if it was less than or equal to 3; there were *n*_high_ = 38 such people). We then transformed the participant-level variable into a dyad-level variable. We categorized the dyads into (a) {high, high} if both individuals in a dyad had a high out-degree centrality, (b) {low, low} if both individuals in a dyad had a low out-degree centrality, and (c) {low, high} if one individual in a dyad had a low out-degree centrality and the other individual had a high out-degree centrality. Of the 1,952 unique dyads with complete fMRI and social-network data, 300 dyads were {high, high} dyads, 702 were {low, high} dyads, and 950 were {low, low} dyads with respect to their levels of objective social disconnection.

**Relating subjective and objective social disconnection**. We found that subjective and objective social disconnection were significantly correlated with one another, such that greater loneliness was associated significantly with a smaller number of friends (with a Pearson correlation coefficient of *r*(61) = –0.337 and a *p* value of *p* = 0.007).

**Supplementary results: Controlling for in-degree centrality**

**Results associating neural similarity with binarized loneliness when we control for in-degree centrality and self-reported demographic traits.** For exploratory purposes, we fit analogous models to those that we described in the main manuscript in relating loneliness with neural similarity when we control for objective social disconnection, friendships between participants, and demographic similarities of the Results section, except that we controlled for individuals’ in-degree centralities instead of their out-degree centralities. We calculated the in-degree centrality of each individual as follows. As we noted in the main manuscript (see “Characterizing subjective and objective social disconnection” section in the Method section), we characterized the social networks of individuals who lived in two different residential communities of first-year students at a large state university in the United States. Using the responses of the individuals, we constructed a directed network for each of the two communities. In each of these networks, a node represents an individual and a directed edge represents one individual nominating another as a friend. For each individual, we calculated in-degree centrality, which counts the number of times that the individual was nominated as a friend by others in the network. We then used a median split of the in-degree centralities to binarize our sample into high-centrality and low-centrality groups. This choice is consistent with recent studies that related neural similarity with behavioral measures (Finn et al., 2018; Leong et al., 2020). We classified participants as part of the high-centrality group if they had an in-degree centrality that was larger than the median (specifically, if it was more than 2; there were *n*_high_ = 23 such people) and into the low-centrality group if they had an in-degree centrality that was less than or equal to the median (specifically, if it was less than or equal to 2; there were *n*_low_ = 40 such people). We then transformed the individual-level binarized in-degree centrality measure into a dyad-level variable. We categorized the dyads into (a) {high, high} if both individuals in a dyad had a high in-degree centrality, (b) {low, low} if both individuals in a dyad had a low in-degree centrality, and (c) {low, high} if one individual in a dyad had a low in-degree centrality and the other individual had a high in-degree centrality.

We fit analogous models as the ones that we described in “Relating loneliness with neural similarity when we control for objective social disconnection, friendships between participants, and demographic similarities” section in the Results section of the main manuscript. Specifically, for each brain region, we fit linear mixed-effects models, with the ISCs in the brain region as the dependent variable and the dyad-level loneliness variable as the independent variable of interest, while controlling for in-degree centrality, friendships between individuals in the dyad, and dyadic similarities in age, gender, ethnicity, and home country as covariates of no interest. The results of these calculations (see Fig. S3) are similar to those that we reported in the main manuscript (see Figs. 2 and 4).


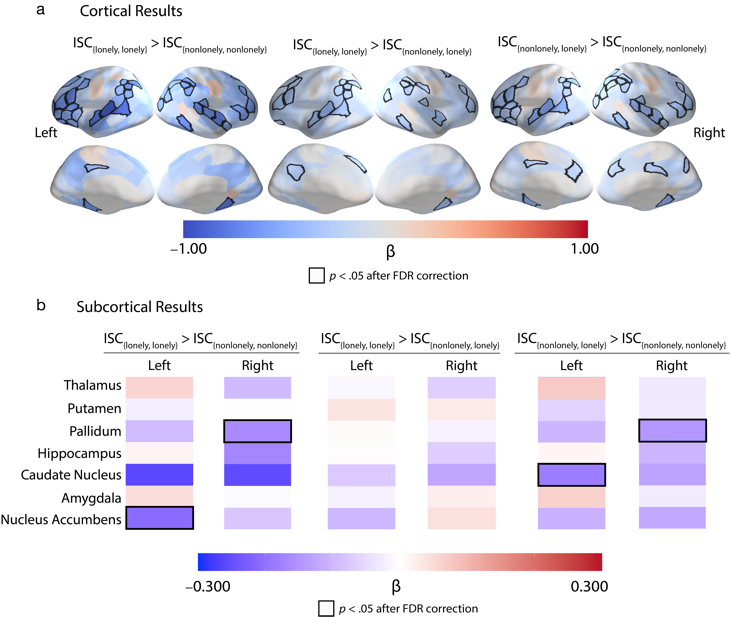


**Fig. S3.** Linking loneliness to idiosyncratic neural responses while controlling for in-degree centrality, demographic similarities, and friendships between participants. **(a)** As in our results in the main manuscript, we observed smaller ISCs in brain regions (including the ventrolateral prefrontal cortex, dorsolateral prefrontal cortex, superior temporal sulcus, inferior parietal lobule, and superior parietal lobule) that are associated with social cognition, shared understanding of events, and friendship in dyads with individuals who were both lonely (i.e., {lonely, lonely}) than in dyads with individuals who were both nonlonely (i.e., {nonlonely, nonlonely}). We observed similar patterns when we compared dyads with two lonely individuals (i.e., {lonely, lonely}) with dyads with one nonlonely individual and one lonely individual (i.e., {nonlonely, lonely}) and when we compared dyads with one nonlonely individual and one lonely individual (i.e., {nonlonely, lonely}) to dyads with two nonlonely individuals (i.e., {nonlonely, nonlonely}). **(b)** The ISCs were smaller in the left nucleus accumbens and right pallidum in dyads with two lonely individuals than in dyads with two nonlonely individuals. The labels “Left” and “Right” refer to the hemispheres of the brain regions that are listed in the left panel. The quantity β is the standardized regression coefficient. Regions with significant associations between loneliness and ISC are outlined in black (using an FDR-corrected significance threshold of *p* < .05).

**Supplementary discussion**

We measured objective social disconnection (i.e., number of friends) 2–3 months after we collected our neuroimaging and loneliness data, and all data were collected during a time period (the first half of the participants’ first year of college) during which both objective and subjective social disconnection may (a) be impacted strongly by situational factors (e.g., whether or not one just moved far away from home) and (b) be subject to greater fluctuations than under other circumstances. Future studies may benefit from simultaneously collecting objective social disconnection, loneliness, and neural data on more established communities. Employing such an approach may also confer greater sensitivity to detect relationships between the relative levels of loneliness of study participants and how idiosyncratically they process the world around them.

References

Avants, B. B., Tustison, N. J., Song, G., Cook, P. A., Klein, A., & Gee, J. C. (2011). A reproducible evaluation of ANTs similarity metric performance in brain image registration. *NeuroImage*, *54*(3), 2033–2044. https://doi.org/10.1016/j.neuroimage.2010.09.025

Esteban, O., Markiewicz, C. J., Blair, R. W., Moodie, C. A., Ayse, I., Erramuzpe, A., Kent, J. D., Goncalves, M., Dupre, E., Snyder, M., Oya, H., Ghosh, S. S., Wright, J., Durnez, J., Poldrack, R. A., & Gorgolewski, K. J. (2019). FMRIPrep: A robust preprocessing pipeline for functional MRI. *Nature Methods*, *16*, 111–116. https://doi.org/10.1038/s41592-018-0235-4

Finn, E. S., Corlett, P. R., Chen, G., Bandettini, P. A., & Constable, R. T. (2018). Trait paranoia shapes inter-subject synchrony in brain activity during an ambiguous social narrative. *Nature Communications*, *9*, 2043. https://doi.org/10.1038/s41467-018-04387-2

Leong, Y. C., Chen, J., Willer, R., & Zaki, J. (2020). Conservative and liberal attitudes drive polarized neural responses to political content. *Proceedings of the National Academy of Sciences, USA*, *117*(44), 27731–27739. https://doi.org/10.1073/pnas.2008530117

Smith, S. M., Jenkinson, M., Woolrich, M. W., Beckmann, C. F., Behrens, T. E. J., Johansen-Berg, H., Bannister, P. R., De Luca, M., Drobnjak, I., Flitney, D. E., Niazy, R. K., Saunders, J., Vickers, J., Zhang, Y., De Stefano, N., Brady, J. M., & Matthews, P. M. (2004). Advances in functional and structural MR image analysis and implementation as FSL. *NeuroImage*, *23*, S208–S219. https://doi.org/10.1016/j.neuroimage.2004.07.051
